# Deep sequencing-based transcriptome profiling analysis of bacteria-challenged *Lateolabrax japonicus *reveals insight into the immune-relevant genes in marine fish

**DOI:** 10.1186/1471-2164-11-472

**Published:** 2010-08-13

**Authors:** Li-xin Xiang, Ding He, Wei-ren Dong, Yi-wen Zhang, Jian-zhong Shao

**Affiliations:** 1 College of Life Science, Zhejiang University, Hangzhou 310058, China; 2 Key lab for Cell and Gene Engineering of Zhejiang province, Hangzhou 310058, China

## Abstract

**Background:**

Systematic research on fish immunogenetics is indispensable in understanding the origin and evolution of immune systems. This has long been a challenging task because of the limited number of deep sequencing technologies and genome backgrounds of non-model fish available. The newly developed Solexa/Illumina RNA-seq and Digital gene expression (DGE) are high-throughput sequencing approaches and are powerful tools for genomic studies at the transcriptome level. This study reports the transcriptome profiling analysis of bacteria-challenged *Lateolabrax japonicus *using RNA-seq and DGE in an attempt to gain insights into the immunogenetics of marine fish.

**Results:**

RNA-seq analysis generated 169,950 non-redundant consensus sequences, among which 48,987 functional transcripts with complete or various length encoding regions were identified. More than 52% of these transcripts are possibly involved in approximately 219 known metabolic or signalling pathways, while 2,673 transcripts were associated with immune-relevant genes. In addition, approximately 8% of the transcripts appeared to be fish-specific genes that have never been described before. DGE analysis revealed that the host transcriptome profile of *Vibrio harveyi-*challenged *L. japonicus *is considerably altered, as indicated by the significant up- or down-regulation of 1,224 strong infection-responsive transcripts. Results indicated an overall conservation of the components and transcriptome alterations underlying innate and adaptive immunity in fish and other vertebrate models. Analysis suggested the acquisition of numerous fish-specific immune system components during early vertebrate evolution.

**Conclusion:**

This study provided a global survey of host defence gene activities against bacterial challenge in a non-model marine fish. Results can contribute to the in-depth study of candidate genes in marine fish immunity, and help improve current understanding of host-pathogen interactions and evolutionary history of immunogenetics from fish to mammals.

## Background

Since it is a representative population of lower vertebrates serving as an essential link to early vertebrate evolution, fish is believed to be an important model in various developmental and comparative evolutionary studies [1-3]. Fish immunogenetics has received considerable attention due to its essential role in understanding the origin and evolution of immune systems [4-6]. Further, it is also beneficial in the creation of immune-based therapy of severe fish diseases. Great progress in bioinformatics and genome projects in model organisms, including human (*Homo sapiens*), mouse (*Mus musculus*), frog (*Xenopus laevis*), chicken (*Gallus gallus*), and zebrafish (*Danio rerio*), has led to the emergence of studies focusing on the identification and characterization of immune-related genes in teleost fish based on comparative genomics. These have provided preliminary observations on fish immunogenetics and evolutionary history of immune systems from lower vertebrates to mammals [7,8]. However, large-scale identification of immune-related genes at the genome or transcriptome levels in fish was seen in limited species (e.g. *Danio rerio*) due to the inadequate number of high-throughput deep sequencing technologies available [9,10]. This is an even more difficult problem in non-model fish species with totally unknown genome sequences.

Recently developed RNA deep sequencing technologies, such as Solexa/Illumina RNA-seq and Digital gene expression (DGE), have dramatically changed the way immune-related genes in fish are identified because these technologies facilitate the investigation of the functional complexity of transcriptomes [11,12]. RNA-Seq refers to whole transcriptome shotgun sequencing wherein mRNA or cDNA is mechanically fragmented, resulting in overlapping short fragments that cover the entire transcriptome. DGE is a tag-based transcriptome sequencing approach where short raw tags are generated by endonuclease. The expression level of virtually all genes in the sample is measured by counting the number of individual mRNA molecules produced from each gene. Compared with DGE analysis, the RNA-Seq approach is more powerful for unraveling transcriptome complexity, and for identification of genes, structure of transcripts, alternative splicing, non-coding RNAs, and new transcription units. In contrast, the DGE protocol is more suitable and affordable for comparative gene expression studies because it enables direct transcript profiling without compromise and potential bias, thus allowing for a more sensitive and accurate profiling of the transcriptome that more closely resembles the biology of the cell [9,13-17]. These two technologies have been used in transcriptome profiling studies for various applications, including cellular development, cancer, and immune defence of various organisms [10,18-29]. However, they have not been used in immunogenetic analysis of marine fish species.

Japanese sea bass (*Lateolabrax japonicus*) is an economically important marine species widely cultured in fisheries worldwide. Various diseases caused by bacterial and viral pathogens plague this species [30]. High mortality is associated with infection with *Vibrio harveyi*, a typical gram-negative pathogen of a wide range of marine animals. Infection results in a variety of vibriosis, a common aquatic animal disease associated with high mortality throughout the world [31]. In *L. japonicus*, *V. harveyi *infection leads to bacterial septicaemia with muscle ulcer as well as subcutaneous and gastroenteritic haemorrhage.

The present study is the first to conduct a transcriptome profiling analysis of *V. harveyi*-challenged *L. japonicus *using RNA-seq and DGE to gain deep insight into the immunogenetics of marine fish. Bacteria-challenged *L. japonicus *is expected to be a model system for studying bacterial immunity in marine fish. Further, a global survey of anti-bacterial immune defence gene activities in marine fish can contribute to the in-depth investigation of candidate genes in fish immunity. Results are also expected to improve current understanding of host-pathogen interactions and evolutionary history of immunogenetics from fish to mammals.

## Results

### Aligning raw sequencing reads to non-redundant consensus

Approximately 34.59 and 33.03 million 75-bp pair end (PE) raw reads (submitted to GEO database, Association No. GSE21721) from the head kidney and spleen tissues of bacteria- and mock-challenged fish, respectively, were generated using Solexa/Illumina RNA-seq deep sequencing analysis. Repetitive, low-complexity, and low-quality reads were filtered out prior to assembly of sequence reads for non-redundant consensus. Using Grape software, reliable reads were assembled into contigs, which were then compared with all PE reads. Overlap of PE reads with two contigs was taken to indicate that the contigs are short segments of a scaffold. Reads were used for gap-filling of these scaffolds to generate final scaffold sequences. Using tgicl and cap3 software programs, scaffold sequences were assembled into clusters that were then analysed for consensus. A total of 150,125 and 140,330 non-redundant consensus sequences, ranging from 100 to 2,000-bp, were generated from each group. Then, consensus sequences were merged for DGE analysis. Removal of partial overlapping sequences yielded 169,950 non-redundant consensus sequences (Table 1). These sequences provide abundant data on healthy and infected conditions, thus allowing for better reference of immune-relevant genes.

**Table 1:** Distribution of non-redundant consensus sequences

Consensus Length (bp)	Total Number	Percentage
100-500	143865	84.65%
500-1000	14313	8.42%
1000-1500	5475	3.22%
1500-2000	2849	1.68%
≥2000	3448	2.03%
Total	169950	100.00%
		
Total bp	57817772	
N50	598	
Mean	340	

N50 = median length of all non-redundant consensus sequences

Mean = average length of all consensus sequences

### Annotation of all non-redundant consensus sequences

**Figure 1:**
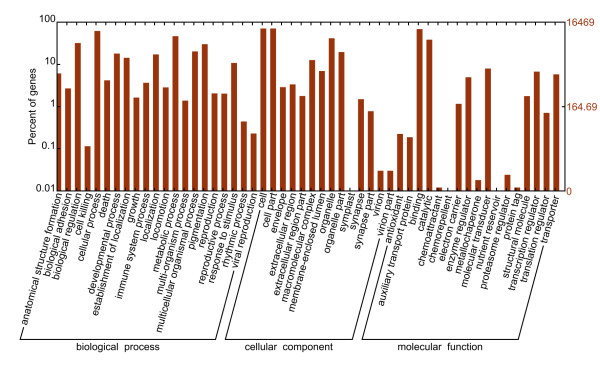
**GO annotations of non-redundant consensus sequences**. Best hits were aligned to the GO database, and 16,469 transcripts were assigned to at least one GO term. Most consensus sequences were grouped into three major functional categories, namely biological process, cellular component, and molecular function.

**Figure 2:**
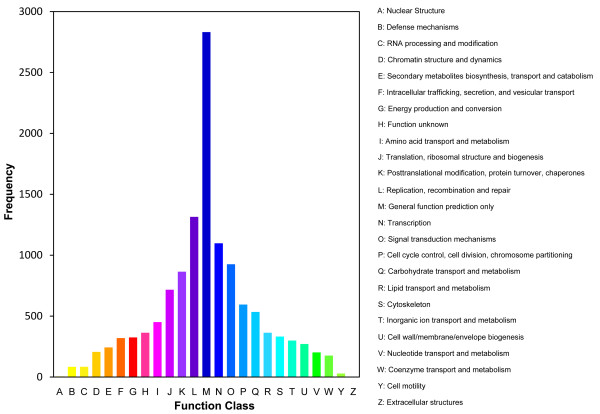
**COG annotations of putative proteins**. All putative proteins were aligned to the COG database and can be classified functionally into at least 25 molecular families.

**Figure 3:**
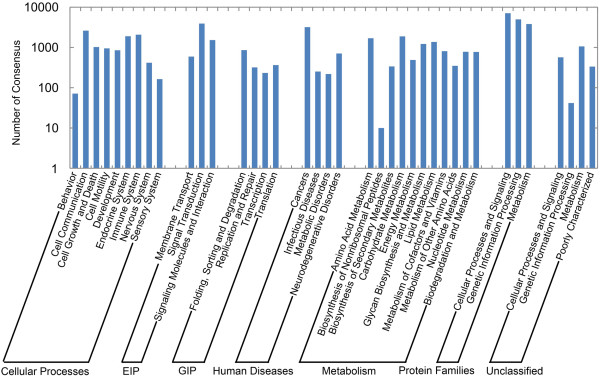
**KEGG categories of non-redundant consensus sequences**. All non-redundant consensus sequences were annotated using KEGG Automatic Annotation Server for pathway information, and about 24,496 consensus sequences were annotated. The categories GIP and EIP stand for genetic information processing and environmental information processing, respectively.

BLASTX and ESTscan software analysis of 169,950 non-redundant consensus sequences revealed that about 48,987 have reliable coding sequences (CDs) [32,33]. CD-containing consensus sequences have high potential for translation into functional proteins and most of them translated to proteins with more than 100 aa. Comparison with the Nr and Swissprot databases revealed that 44,842 consensus sequences had good comparability with known gene sequences in existing species. Annotation of the 44,842 sequences using GO and COG databases yielded good results for approximately 16,469 consensus sequences and 9,545 putative proteins [34,35]. GO-annotated consensus sequences belonged to the biological process, cellular component, and molecular function clusters and distributed among more than 50 categories (Figure 1), including biochemistry, metabolism, growth, development, apoptosis, and immune defence. Similarly, COG-annotated putative proteins were classified functionally into at least 25 molecular families, including cellular structure, biochemistry metabolism, molecular processing, signal transduction, gene expression, and immune defence, that correspond to the categories observed in GO analysis (Figure 2). The KEGG database was used to analyse potential involvement of the consensus sequences in cellular metabolic pathways (Figure 3) [36]. Among the 44,842 consensus sequences, 24,496 can be grouped into seven categories comprised of 219 known metabolic or signalling pathways, including cellular growth, differentiation, apoptosis, migration, endocrine, and various immune-relevant signalling or metabolic pathways (Table 2).

**Table 2:** Annotation of non-redundant consensus sequences

Database	Number of annotated consensus sequences	Percentage of annotated consensus sequences
Swissprot	37544	76.64%
Nr	36935	75.40%
GO	16469	33.62%
KEGG	24496	50.01%
COG	9545	19.48%

All 48,987 CD-containing consensus sequences generated by ESTscan were annotated though Swissprot, Nr, GO, KEGG, and COG databases.

### Annotation of immune-relevant genes and pathways

To gain deep insight into the molecular biology of immune systems in *L. japonicus*, the immune-relevant genes, metabolic and signalling pathways were analysed. Approximately 2,673 consensus sequences were found to be homologous to known immune-relevant genes in other species (Table 3, Additional file 1, Table S1), including the most important elements of innate and adaptive immunity, such as pattern recognition receptors (mannose binding proteins, scavenger receptors, Toll-like receptors, and lipopolysaccharide-binding proteins), inflammatory cytokines and receptors (IL-1β, IL-10, IL-12, IL-6, IL-8, TNF-α, IFN-γ, TGF-β, VEGF, IL-1, and TNF receptors), immunoglobins (IgM and IgD), transcriptional factors (NF-kB, Ik-B, AP-1, transcription factor 4, chorion specific transcription factor, and transcriptional enhancer factor 5), complement components (C1q, C1r, C1 s, C2, C4, MASP, Bf, Df, and CR1-5), leukocyte differentiation antigens (CD3, CD4, CD8, and CD80/86), antigen presenting and processing molecules (MHC I, MHC II, TAP1, TAP2, TAP2B, proteosome subunit, PA28α/β, and constant chain protein li), regulatory molecules involved in immune cellular proliferation, differentiation, and apoptosis (growth differentiation factor 11, proliferation associated protein 2G4, apoptotic peptidase activating factor 1, and anti-apoptotic protein NR13), and other molecules involved in immune response (hepcidin, lectin, lysozyme, antimicrobial peptide NK-lysin, chemokines, ferritin, RAG1, and RAG2).

**Table 3:** Immune-relevant genes/homologues in *L. japonicus*

Putative Gene Catalogs	Consensus Number	Homologs Number in other species	Putative Gene Catalogs	Consensus Number	Homologs Number in other species
***Pattern Recognition Genes***			***T-cell and B-cell Antigen Activation***		
TLR (1-9,13,14,18,21,23)	44	17	TCR (α,β chain)	6	4
scavenger receptor	19	11	BCL (2,3,6,7,9,10,11) and relevant	44	19
Mannose receptor	49	26	Immunoglobulin and relevant	52	32
MBL	13	9	CD (2-6,8,9,22,40,79,81,97,99,154,166, 226,276)	47	29
NOD (1,2)	7	5	BLNK	5	3
C-type lectin (4,6,7,9-12)	36	26	AICDA	3	2
TOLLIP	1	1	CBLB	7	4
LPS-binding/anchor protein	19	3	DOCK2	4	2
HMG (A,B,2L1,20A)	8	6	DRAM	1	1
LRR-containing proteins	168	74	Glomulin	2	2
NALP (1,3,4,6,9,12,13)	21	8	HDAC (1-8,10,11)	40	23
PGRP	1	1	GALNAC4S-6ST	5	3
BTK	2	2	KLF (1-4,6-9,11-13)	29	15
Fibrinogen	4	4	IGBP1	1	1
***Complement System***			HLA	1	1
C1 (q,r,s,t),C2-C7,C9,C10	40	32	JAG (1,2)	37	6
Complement factor (B,H,I,properdin, DAF)	22	11	KI13B	17	1
Complement receptor (1,2,5)	13	8	LAG	1	1
MASP	4	3	NCK	7	5
RGC	2	2	Homeobox protein (NKX)	4	3
***Inflammatory Cytokines and Receptors***			PAWR	3	3
IL (1,3,8,10-12,15,16,27,34) and relevant	34	17	Protein kinase C δ	6	3
ILR (1-3,6,7,10,12,15,17,18,20-22,31) and relevant	48	31	Prolactin receptor	5	2
IFN	3	2	RAG (1,2)	3	2
IFN-induced proteins and relevant	59	27	RGS (1-5,7-9,11,12,14,16,18-20)	47	23
IFN (α,β,ω) receptor	2	1	SFTPD/SP-D	1	1
IFN-related regulator	31	16	TYK2	1	1
CRLF (1,3)	3	2	VAV oncogene family (1-3)	13	6
EBI 2	5	2	WAS proteins	23	11
FND (3,C)	12	4	WWP	2	2
TTN/TITIN	5	2	***Antigen Processing and Regulators***		
CXCL(6,12,14),CXC,CXCR(1-5,7)	20	17	MHC I/II	14	5
CCL(2-4,7,14,16,19,C11b),CCR(2-9,11)	33	26	PSMA (1-7)/B/C/D/E(2-4)	93	63
CX3CR	1	1	Legumain	5	3
XCL(1),XCR(1,1a)	6	3	ITCH	5	2
Chemokine receptor-like	4	2	RFX5	4	1
CFLAR	1	1	TAPBP	3	2
EGF	125	50	ASPP (1,2)	24	4
FAM	15	8	ICAM2	1	1
FGF	23	14	RAC1	3	1
FLT3/CD135	2	2	AGT	4	2
GATA (1-3,5,6)	14	8	CDC (2,5-7,16,20,23,27,42,A)	23	15
GDF	3	3	CDK-inhibitor (1-3)	9	8
GFI-1/-1B	13	3	CTLA4	1	1
GPR44	4	2	***Other Genes Related to Immune Cell Response***		
Inhibin β	1	1	CD (33,34,44,45,48,83,151,302)	10	8
Integrin α/β	89	38	Apolipoprotein A	2	2
LIFR	5	3	HELLS	1	1
Lipoteichoic acid	1	1	IMPDH/IMDH	4	3
MAF	2	2	AP-1	20	15
MIF	3	2	c-FOS	2	2
PCGF (1-3, 5, 6, A)	8	6	EGR	11	5
SCYE1/MCA	3	2	ELK4	1	1
SDF2	2	2	IKKA/CHUK, IKKB	8	2
TGF A/B(1-3)	10	9	JAK (1,2)	11	6
TNF and TNR	54	38	JUN oncogene	11	6
Thrombopoietin	1	1	MALT	5	2
VEGF (A,C,D)	5	4	NFAT	32	12
VEGF receptor (1-3)	17	8	NF κ-B inhibitor proteins	11	4
***Adapters, Effectors and Signal Transducers***			PPM	33	14
ASB (1-3,5-8,12,13)	26	14	RIPK (1-3,5)	7	5
CRADD	2	1	SLC20A-1/2	6	4
DEDD	4	2	Transcription factor Sp and SPT	21	18
EIF 2-α kinase 2	7	2	TANK-binding kinase	9	5
FADD	1	1	TBX	12	7
Heme oxygenase	4	2	TMED (1-5,7-9,A)	12	10
HTR (5A,5B)	22	4	TRIM (1-3,7-9)	20	8
IRAK (1-4)	7	6	Antimicrobial peptide	2	2
CSF	12	5	Co-chaperone	4	3
Myd88	2	1	Ferritin	1	1
NR2C	6	4	Hepcidin	1	1
PEA15	2	1	HSP (7,13,70-72,74,B)	35	20
PELI (1-3)	21	6	Lysozyme	4	3
PHLA (1-3)	4	3	Macroglobulin	9	6
PPARA	2	2	Microtubule-associated	109	45
REL	1	1	Nitric oxide synthase	4	3
RHOA	3	1	Proteolipid protein	1	1
RIP	2	2	Prohibitin	3	2
SARM1	8	2	Selenoprotein	22	11
SIGIRR	2	2	ABCF	8	7
SMAD (1-7,9)	33	16	CAMP	13	4
SOCS (1-7)	18	8	CARD	5	3
TICAM1	1	1	CEBP	7	5
TIRAP	1	1	DMBT	6	4
TRADD	1	1	PTAFR	1	1
TRAF (2-7)	10	7	Protein S100-A	5	4
TANK	2	2	UCRP/CRP	1	1
UBE	47	25	CYBB	1	1
STAT	21	7	Caspase (1-3,6-9)	18	13
NF κ	15	4	Adenosine receptor A (2a,2b)	11	7
MAPK (1-3,5-9,11-15)	47	29	Apoptotic relevant	13	6
MAP2K4(JUKK1)	2	2	IRG1	4	2
MAP3K (2-9,11,14,15)	35	18	Stress response protein	22	6
MAP3K 7-interacting protein (TAB)	4	4	WSC (1,2,4)	8	3
JNK	5	4	OSGI2	1	1

Putative genes/homologues were confirmed based on their similarity to immune-relevant genes in other vertebrate species as predicted by BLAST. Cutoff E-value was set to 1e-10 during BLAST analysis. Characterization of the putative genes is presented in Additional file 1, Table S1.

**Figure 4:**
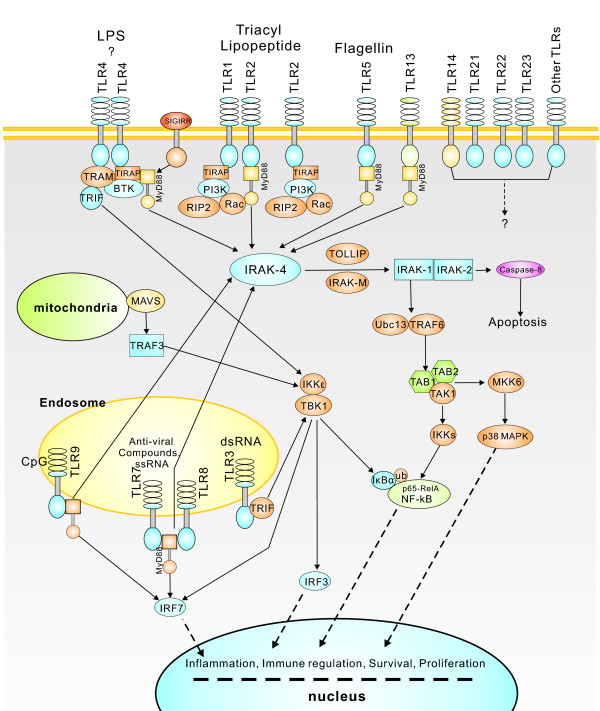
**Putative TLR signal pathway**. Putative TLR signal pathway of *L. japonicus *was constructed based on knowledge of TLR signalling in mammalian species. However, most interactions have to be confirmed experimentally.

**Figure 5:**
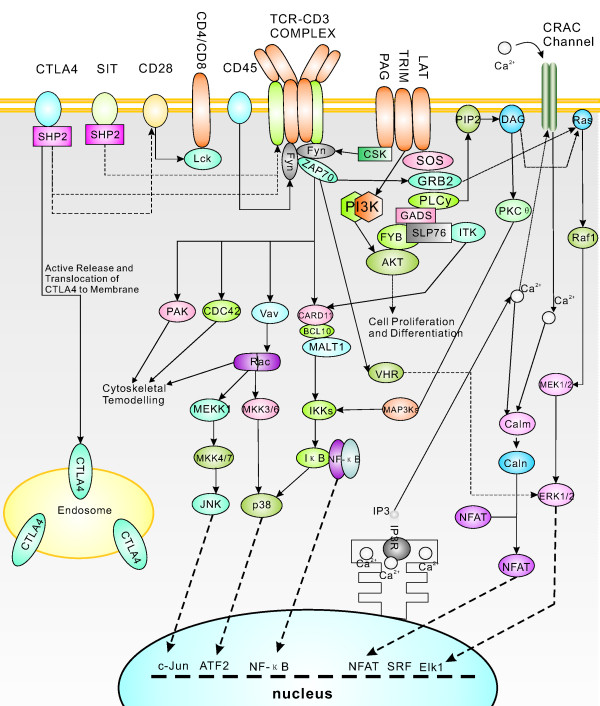
**Putative TCR signal pathway**. Putative TCR signal pathway of *L. japonicus *was constructed based on knowledge of TCR signalling in mammalian species. However, most interactions require experimental confirmation.

KEGG analysis revealed that approximately 2,082 consensus sequences were significantly enriched in various known immune-relevant metabolic or signalling pathways (Figure 3). These suggest a considerable conservation of immune-relevant genes and pathways between *L. japonicus *and mammals. Conserved genes and pathway members might include Toll-like receptors (TLR1-9 and TLR13) and corresponding adaptors in mammals (MyD88, TRAM, FADD, CASP8, IRAK4, TRAF6, TAK1, MKK4/7, JNK, and AP-1) and in other fish species (TLR5a/b, TLR14 and TLR21-23) (Figure 4); T cell receptors (TCRα/β) and corresponding signalling transducers (Zap70, Lyn, Fyb, SHP1, CD3, CD28, CD45, LCK, PAK, CDC42, Vav, CARD11, PAG, ITAM, LAT, PIP2, SLP-76, MAP3Ks, IKKs, CBL, NCK, LAT, GRB2, CARMA1, NFAT, MALT1, GRB2, JNK, MKKs, PAK, Ras, Raf1, and MEKK1) (Figure 5); B cell receptors and downstream factors (B-cell antigen receptor complex-associated protein α/β chain, CD81, CD22, and BCL-10); key molecules involved in antigen presenting and processing pathways (MHC class I and II, Hsp70, Hsp90, and Calnexin); members of complement and coagulation pathways (classical pathway members C1q, C1r, C1 s, C2, and C4, MBL pathway members MBL and MASP, alternative pathway members Bf and Df, and complement receptor type 1); and members involved in FcεR I signalling pathway (PI3K and SYK), leukocyte migration (lymphotactin, Rho GTPase-activating protein 5, RhoH, integrin alpha-M, and leukaemia inhibitory factor receptor), and natural killer-mediated cytotoxicity (natural killer cell receptor 2B4, natural killer cell protease 1, natural killer cell enhancement factor, natural killer-tumour recognition sequence, and natural killer cell stimulatory factor 2). Furthermore, a number of consensus genes involved in cellular adhesion, energy production, and amino acid (arginine and praline) metabolisms were also conserved between fish and mammals. These genes are indirectly related to immune responses in mammals. For instance, L-arginine metabolism has been proven to be related to phagocytosis of macrophages, which eventually led to the discovery of NO signalling molecule [37]. Thus, the involvement of these consensus genes in metabolic pathways provides the basis for further identification of the biological functions of candidate genes in fish immune responses.

### Digital gene expression profile analysis after bacterial challenge

**Figure 6:**
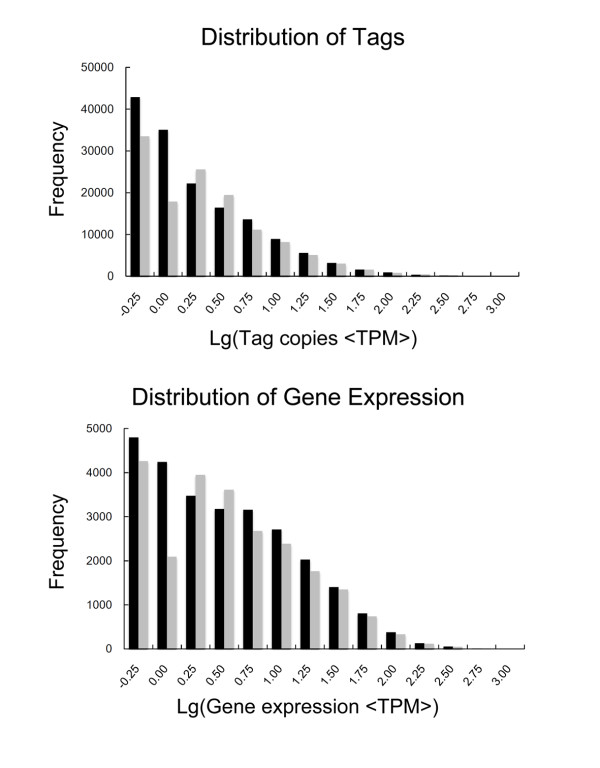
**Distribution of tags and gene expression between experimental and control groups**. The black and gray columns indicate the distribution of tags and gene expression in bacteria- and mock-challenged groups, respectively. The distribution of tags matches the distribution of genes for both groups. Furthermore, an increase in tags or gene expression is accompanied by a decrease in the frequencies of tags or genes.

Solexa/Illumina DGE analysis was performed to identify the genes involved in *L. Japonicus *response to bacterial challenge [12]. A total of 3.44 and 3.22 million raw tags (submitted to GEO database, Association No. GSE21712) of the mRNAs extracted from head kidney and spleen of the mock- and bacteria-challenged groups, respectively, were identified by base calling [38,39]. After transformation of raw sequences into clean tags by data-processing steps using bio-perl scripts, approximately 0.33 and 0.27 million high quality non-redundant tags were obtained in both groups (Figure 6). Gene annotation was performed by tag mapping analysis using the 169,950 non-redundant consensus sequences from RNA-seq-based transcriptome analysis as reference transcript database. Results showed that 71.41% and 74.53% of all distinct tags can be mapped to the entire reference database (sense or anti-sense) in both groups. Out of the 26,394 sense strands and 23,790 anti-sense strands detected in the mock-challenged group, about 36,782 (75.1%) sense or anti-sense strands were mapped by the tags. In contrast, about 34,840 (71.1%) sense or anti-sense genes were mapped out of the 23,359 sense strands and 21,046 anti-sense strands in the infected group (Figure 6). Among the detectable expressed consensus sequences, 9,643 genes had successful annotations. Mapping results are summarized in Additional file 2, Table S2 and Additional file 3, Figure S1.

**Figure 7:**
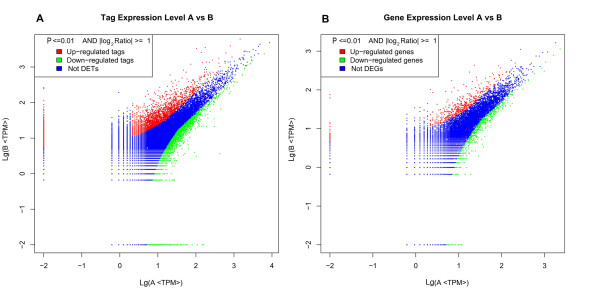
**Differential expression analyses of tags and consensus sequences by DGE**. The expression level for each tag and consensus was included in the volcano plot (Figures A and B, respectively). 'Not DETs' and 'Not DEGs' indicate 'not detected expression tags' and 'not detected expression genes', respectively. For Figures A and B, the x-axis contains Log_10 _of transcript per million of the bacteria-challenged group and the y-axis indicates Log_10 _of transcript per million of the mock-challenged group. Limitations are based on P < 0.01, and the absolute value of Log_2 _(B/A) is greater than 1.

**Figure 8:**
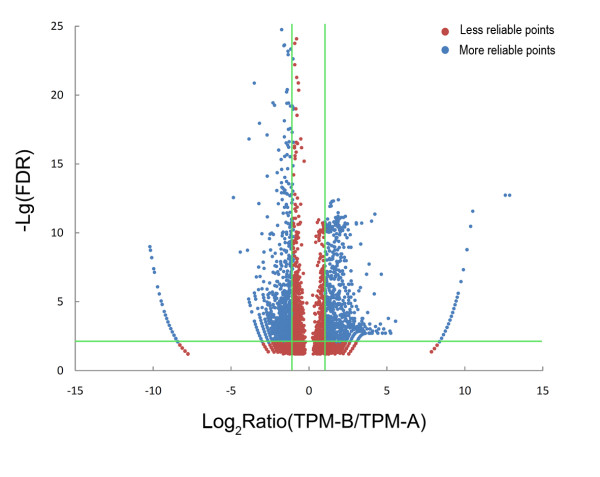
**Volcano plot of differentially expressed consensus sequences**. For every consensus, the ratio of expression levels in the bacteria-challenged group over that in the mock-challenged group was plotted against the -log error rate. The horizontal line indicates the significance threshold (0.01 FDR), and the vertical lines indicate the twofold change threshold.

Strict Bayesian algorithm was used in differential DGE analysis in order to consider the differences in library size for differential selection between the two differentially expressed gene libraries [40]. Soap2 software was used to map all measured tags to the corresponding assembled consensus sequences [41]. P ≤ 0.01 and absolute value of log_2_Ratio≥1 were used as the threshold of significant differences in gene expression. Approximately 1,224 CD-containing consensus sequences mapped by 19,548 differentially expressed tags exhibited significant differences after bacterial challenge. Among them, about 376 consensus sequences were significantly up-regulated (including 61 most over-expressed sequences that increased by more than 100-fold), while 848 consensus sequences were significantly down-regulated (including 10 most down-regulated sequences that decreased by 10-100-times) (Figure 7, Additional file 4, Table S3). The distribution of the significant changes detected is illustrated in a volcano plot (Figure 8), where the statistical significance of each consensus was plotted against fold change. Sequences with the highest average differences between the bacteria- and mock-challenged groups (far right and left of the plot) also had the smallest false discovery rate (FDR) values. Analysis of the CD-containing consensus sequences revealed that they all had FDR values less than 0.1, with the highest value being 0.063.

Functional annotation of consensus sequences was performed to define the differentially regulated sequences more precisely. Majority of the 1,183 sequences annotated were related to factors involved in various immune-relevant pathways, such as TLR pathways (TLR1/3/13/18/21, BTK, IRAK1, IP2, NR2C2, and UBE2n), TCR signalling pathways (TCR beta chain, Zap70, LCK, SHP1, CARMA1, Vav, NFAT, GRB2, MALT1, NCK, and Raf1), antigen presenting and processing relevant pathways (MHC class II transactivator, CASP1, and CASP8), TGF-β signalling pathway (TGF-β receptor type-2, Serine/threonine-protein phosphatase 4, serine/threonine kinase receptor, BMPs, and SMAD7), and various inflammatory cytokines and receptor relevant pathways (IL18R, IL12R β-2 chain, C-C motif chemokine 7, C-C motif chemokine 19, TGFβ-1-induced transcript 1 protein, TNR superfamily member 1A, and TNFα-induced protein 2). There were annotations in several other biological processes that may indirectly participate in immune response, such as the cell cycle; DNA replication, transcription, and translation; metabolisms of carbohydrates, amino acids, and lipids; and activation of ATPase family members, transcription elongation factor B, membrane transport protein, NADH dehydrogenase, NAD kinase, nucleolar protein 6, tyrosine protein kinase, ribosomal protein L32, nuclear receptor, and replication initiator 1. Among the 61 most over-expressed transcripts and the 10 most down-regulated transcripts, enrichments of factors involved in metabolic or signalling pathways that have not been linked to immune responses previously, such as cytoskeleton regulation, calcium signalling pathway, MAPK signalling pathway, aimnocyl-tRNA biosynthesis, and methionine, glutamate, and aminosugar metabolism, were detected. Highly responsive consensus sequences are shown in Table 4.

**Table 4:** Representatives of putative immune relevant genes/homologs as predicted by DGE

Catalogs	Consensus ID	Putative Identification	Fold Change
***Pattern Recognition Proteins***			
TLR and relevant	3365	TLR 1	-6.45
	8296	TLR 21	-374.00
Mannose receptor	158882	Macrophage mannose receptor 1	-2.94
NOD	117946	NOD protein 2	+4.25
C-type lectin	36609	Collectin-12	+3.69
HMG	3367	HMG AT-hook 1	-218.00
LRR-containing proteins	128723	F-box/LRR protein 14	+5.87
	5434	F-box/LRR protein 20	-2.35
	36369	F-box/LRR protein	-249.00
	3391	LRR-containing protein 68	-6.14
	39099	LRR flightless-interacting protein 2	+2.32
NALP	62330	NALP 3	-2.04
	43979	NALP 6	-2.47
	719	NALP 12	-312.00
	14950	NALP 13	-3.77
BTK	37665	BTK	-249.00
***Complement System***			
C1	39636	C1q-like protein 4	+5.03
Complement factor	58617	Complement factor H-related protein 3	-2.55
***Inflammatory Cytokines and Receptors***			
IL, ILR, and relevant	7183	IL6R subunit β	-3.06
	4828	IL7R subunit α	-218.00
	29	IL10R α chain	+265.00
	8308	IL12R β-2 chain	-7.09
	70266	Putative IL17R E-like	+2.23
	9160	IL18R	-2.33
	36378	IL18R accessory protein	-2.96
	67818	IL31RA	-2.38
	4392	IL enhancer-binding factor 3	-4.29
IFN and relevant	34	similar to IFN-inducible protein Gig1	-3.76
	39770	IFN-induced helicase C domain-containing protein 1	-2.15
	39343	IFN-induced GTP-binding protein Mx	+2.22
	42748	IFN-induced transmembrane protein 2	-8.97
	41016	IFN regulatory factor 4	-5.98
	43856	IFN regulatory factor 8	-6.14
CXCR	41529	CXC chemokine receptor 5	-2.59
CCL	71546	C-C motif chemokine 19	+2.20
XCR	70282	XC Chemokine receptor 1	+4.03
EGF	8053	SNED	-312.00
FAM	37053	Protein FAM3C	-2.35
FLT	9012	FLT3 ligand cytokine receptor	-405.00
GATA	42892	GATA-binding factor 2	-15.43
GFI	6300	Zinc finger protein GFI-1	+6.58
	16357	Zinc finger protein GFI-1B	-2.01
Integrin	169765	Integrin α-3	+298.00
PCGF	36691	PCGF 2	-2.54
TGF	34228	TGF β-1-induced transcript 1 protein	-4.02
Thrombopoietin	8606	Thrombopoietin receptor	-4.19
TNF and TNR	167272	TNF α-induced protein 8-like protein 2	-6.38
	36673	TNR 1A	-405.00
	2890	TNR 12A	-281.00
	9356	EMILIN 1	-2.40
***Adapters, Effectors, and Signal Transducers***			
ASB	71979	ASB2	-5.04
DEDD	1437	DEDD	-3.40
Heme oxygenase	71817	Heme oxygenase	+2.48
CSF	50449	Macrophage CSF 1 receptor 1	-2.09
	11081	Macrophage CSF 1 receptor 2	-218.00
	49226	Granulocyte CSF receptor	-3.09
NR2C	42918	NR2C 2-associated protein	+4.22
SMAD	42078	SMAD 1	-437.00
	42615	SMAD 5	-405.00
TRAF	42729	TRAF7	-2.27
	70242	TANK	-2.11
UBE	2069	UBE E2C	+2.07
	9613	UBE E2H	-2.42
NF κ	1073	NF κ-A	+3.18
MAPK	68093	MAPK 3	-9.45
	70484	MAPK 6	-312.00
MAP2K	37130	MAP2K4	-10.39
MAP3K	38157	MAP3K3	-3.26
	7280	MAP3K4	-4.92
***T-cell and B-cell Antigen Activation***			
CD	60890	CD2	+2.81
	70094	CD22	+4.10
	4032	CD40 (TNR5)	-343.00
	26107	CD79B	-2.48
DOCK2	4848	DOCK2	-2.54
RAG	36208	RGA2	-6.39
RGS	905	RGS12	-2.60
TYK2	9722	Non-receptor TYK2	-2.11
VAV oncogene family	9761	VAV2	-343.00
***Antigen Processing and Regulators***			
MHC I/II	65343	MHC II transactivator	-218.00
PSM	115644	PSMB3	+3.06
	5004	PSMC2	+2.71
	42169	PSMC6	+3.39
	208	PSMD3	-2.05
	7477	PSMD8	+5.29
TAPBP	10433	TAPBP	-281.00
RAC	36540	RAC1	-2.72
CDC	70253	CDC 16	+2.78
	55784	CDC 42	+331.00
CDK-inhibitor	43078	CDK 4 inhibitor D	+2.58
***Other Genes Related to Immune Cell Response***			
AP-1	41196	AP-1 complex subunit σ-2	+4.03
	10356	AP1 subunit mu-1	+2.05
JAK	818	JAK2	-312.00
MALT	21139	MALT protein	-6.14
PPM	365	Protein phosphatase 1F	-2.14
RIPK	1908	RIPK1	-7.88
TMED	8436	TMED2	-3.18
Hepcidin	56643	Hepcidin	+6.10
HSP	37584	HSP 71	-2.96
Lysozyme	51	Lysozyme g	+2.18
Macroglobulin	2234	α-2-macroglobulin	+3.31
Microtubule-associated	12594	EMAP 3	-249.00
Proteolipid protein	38379	Proteolipid protein 2	-3.60
Prohibitin	70061	Prohibitin-2	-7.09
Selenoprotein	80124	Selenoprotein K	+3.19
ABCF	3906	ABCF 2	-3.49
CARD	1588	CARD 11	-3.08
Caspase	42397	Caspase 1	-2.05
	31546	Caspase 8	-5.07
Stress response protein	44158	Stress response protein NST1	-2.15

Limitations of all differentially expressed genes are based on P ≤ 0.01. The absolute value of Log_2_Ratio≥1 and FDR≤0.05 showed that the genes were significantly altered after bacterial challenge. The cutoff E-value was set to 1e-10 during the gene annotation process. The absolute value of "Fold Change" means magnitude of up- or down-regulation for each gene/homolog after bacteria challenge; "+" indicates up-regulation, "-" indicates down-regulation.

### Putative novel immune/stress response genes

Among the differentially expressed transcripts, more than 1,183 transcripts were well annotated, whereas approximately 41 transcripts had low sequence homology to known sequences in public databases, suggesting that they might be putative novel immune-relevant genes in *L. japonicus *involved in the response to bacterial challenge. Among these novel sequences, 13 were differentially regulated by more than 100-fold, implying that they were strongly infection-responsive genes. ProDom analysis identified one HSP domain- and one protein kinase domain-containing sequence [42]. SignalP and TMHMM programs distinguished 24 sequences with signal peptides or transmembrane domains, suggesting that they are cytokines and transmembrane proteins, respectively [43,44]. Observations presented in Additional file 5, Table S4 can provide guidance for further identification. In-depth functional studies of these novel sequences may benefit the exploration of potential marine fish-specific immune-relevant genes for application in the control of fish diseases.

### Experimental validation of consensus sequences

To validate the integrity of RNA-seq results, representative consensus sequences with complete encoding regions, such as hepcidin, Myf5, SNARE, and two IL-8-like CXC chemokine family members, were selected for experimental cloning and sequencing analyses by RT-PCR (Additional file 6, Table S5). All experimentally examined genes matched the RNA-seq-generated sequences perfectly. One of the two IL-8-like CXC chemokines was newly discovered by this study. The two IL-8-like CXC chemokine family members were identified through phylogenetic analysis. Both sequences conserved the four cysteine residues (C_46_-C_48_-C_74_-C_91_) that are the hallmarks of IL-8 CXC chemokines and can be found throughout the vertebrate IL-8 family (Additional file 7, Figure S2 and Additional file 8, Figure S3). This demonstrates the reliability of RNA-seq results and indicates the necessity for further identification of immune-related genes in *L. japonicus*.

## Discussion

The transcriptome is the complete repertoire of expressed RNA transcripts in a cell. Its characterization is essential in deciphering the functional complexity of the genome and in obtaining a better understanding of cellular activities in organisms, including growth, development, disease, and immune defence. The definition of the transcriptome has long been a challenging task. Traditionally, global gene expression analysis has relied mostly on several approaches, including RNA hybridisation on high-density arrays, whole-genome tiling arrays, expressed sequence tag (EST), serial analysis of gene expression (SAGE), and SAGE-derived technologies, which include massively parallel signature sequencing (MPSS) and polony multiplex analysis of gene expression. However, these approaches have several inherent limitations. For example, the array-based approaches allow detection of specific sequences only and capture the transcriptome while ignoring splice-junction information or alternative splicing events. The EST approach provides only partial sequences of individual cDNA clones, is sensitive to cloning biases, and is associated with high costs and difficulties in data analysis. SAGE and MPSS are also costly and cannot be used for splicing events [9]. Thus, the newly developed Solexa/Illumina RNA-seq and DGE high-throughput deep sequencing approaches have dramatically changed how functional complexity of the transcriptome can be studied. These approaches overcome many of the inherent limitations of traditional systems, making the detection of alternative splicing events and low-abundance transcripts possible. They have been applied recently to several species, such as yeast, *Arabidopsis*, *Chlamydomonas*, Zebrafish, *Drosophila*, *Caenorhabditis*, and human, for different purposes [9,10,18-29].

In this study, the transcriptome profile analysis of bacteria-challenged *L. japonicus *was conducted through these two approaches in an attempt to gain deep insights into the immunogenetics of a marine species. As expected, a large set of transcriptional sequences with complete or differing lengths of encoding regions was generated. KEGG analysis showed that more than 52% of transcripts are enrichment factors involved in approximately 219 known metabolic or signalling pathways, including cellular growth, differentiation, apoptosis, migration, endocrine, and immune system processes. Further, more than 8% of transcripts represent novel fish-specific genes that have never been described previously. Detailed analysis of immune-relevant genes and pathways showed that more than 2,673 transcripts are homologous to known immune-relevant genes, whereas approximately 2,082 transcripts can be enriched in various immune-relevant metabolic or signalling pathways. Challenging the fish with *V. harveyi *resulted in large alterations of the host transcriptome profile, including significant up- or down-regulation of 1,224 transcripts, among which 41 sequences might be novel immune-relevant genes in fish. In addition, several other biological processes that have not been linked to host immunity before, such as the metabolism of carbohydrates, amino acids, and lipids; activation of ATPase, NADH dehydrogenase, NAD kinase, and tyrosine protein kinase; and up-regulation of nuclear receptors, replication initiators, and ribosomal proteins, were found to be dramatically involved in host immune response. These significantly regulated transcripts might represent strong infection-responsive genes in *L. japonicus*, and reflect a number of immune activities during fish defence against bacterial challenge. The transcriptome profiling data sets obtained in this study provide strong basis for future genetic research in marine fish and support further in-depth genome annotation in vertebrates. Future molecular and functional characterisation of infection-responsive genes could lead to global identification of immune-relevant genes and infection markers in marine fish.

At present, transcriptome analysis in fish relies mainly on the EST approach [45,46]. Although there have been an increasing number of ESTs sequenced in a large number of libraries in various fish species, including rainbow trout (*Oncorhynchus mykiss*), Atlantic salmon (*Salmo salar*), medaka (*Oryzias latipes*), and zebrafish (*Danio rerio*), the immune-relevant transcriptional profiling data sets obtained from fish are still insufficient. Recently, DGE- and microarray-based transcriptome profiling studies performed in zebrafish revealed that zebrafish and its developing embryo are useful *in vivo *models for the identification of host determinants of responses to bacterial infection [9,10]. However, transcriptional information on immune responses to infection in a non-model marine fish remains elusive. Therefore, the large set of immune-relevant genes and their role in responses to bacterial challenge in *L. japonicus *presented in this study may largely improve knowledge on fish immunogenetics in other analytical systems. The present study also demonstrates the advantages of new deep sequencing approaches for gene discovery, thus providing new leads for functional studies of candidate genes involved in host-bacteria interactions. The RNA-Seq and DGE analyses conducted in this study were found to complement each other well. RNA-Seq was very effective in unravelling transcriptome complexity, and can detect a large set of genes, including numerous low-expressing genes or novel genes. DEG data can be merged with RNA-Seq data sets, indicating an affordable method for comparative gene expression study. Thus, RNA-Seq was initially performed in this study to provide strong reference transcriptome database for subsequent DGE analysis.

Emerging hallmark components and the cells necessary for innate and adaptive immunity in higher vertebrates have been identified in fish [47,48]. This was the basis for the widely accepted notion that innate and adaptive immunity was established in teleosts about 470 million years ago. However, the exact molecular and cellular basis of immune systems in teleosts remains poorly understood. The precise regulatory mechanisms underlying the innate and adaptive immunity of teleosts remain vague due to the limited immune-relevant genetic information available in fish. The present work on the definition of high-throughput transcriptome data set of the immune system of *L. japonicus *may contribute greatly to better understanding of the molecular and cellular activities involved in fish immunity. Results unexpectedly showed that the fish immune system is more complex than previously thought. On one hand, the substantial amount of immune-relevant genes involved in metabolic and signalling pathways and the induction of genes encoding cell surface receptors, signalling intermediates, transcription factors, and inflammatory mediators show a clear conservation of mechanisms detected in other vertebrate models, including humans. On the other hand, a large set of novel immune response genes and infection markers that have never been linked previously to immune responses in other vertebrate systems was identified in *L. japonicus*, indicating the existence of numerous fish-specific immune activities during early vertebrate evolution.

For instance, the TLR family is the most important class of pattern recognition receptors that play crucial roles in mediating immune responses to pathogenic microorganisms [8,49,50]. Triggering of TLRs by ligands leads to the recruitment of adaptor proteins, resulting in the activation of a range of transcription factors, such as NF-κB, activator protein 1 (AP-1), and IFN regulatory factors (IRFs), through distinct signalling pathways. This eventually leads to the downstream activation of proinflammatory cytokines and receptors, such as IFN-α/β, TNF-α, IL-2, IL-6, IL-8, IL10, CD40, CD86, and MIP1α. To date, 13 TLRs (TLR1-13), at least five adaptor proteins (MyD88, Mal/TIRAP, TIR domain-containing adaptor protein, TRIF/TICAM1, TRAM/TICAM2, and SARM), and numerous downstream effectors have been described in mammals and humans. In the present study, a series of TLRs and corresponding adaptor proteins and downstream effectors were identified in *L. japonicus*. The identified TLRs include the majority seen in mammals and humans (TLR1-13), and four TLRs (TLR14, TLR18, TLR21, and TLR23) seen in fish species. Adaptor proteins and downstream effectors identified include the majority known in mammals and humans, including MYD88, BTK, TOLLIP, FADD, HMGB1, HRAS, HSPD1, CASP8, MAPK8IP3, PELI1, RIPK2, SARM1, TICAM2, TIRAP, EIF2AK2, IRAK1, IRAK2, MAP3K7, MAP3K7IP1, NR2C2, PPARA, PRKRA, TRAF6, UBE2N, and UBE2V1. These adaptor proteins and downstream effectors have been found to be well enriched in various known TLR signalling pathways. Downstream transcriptional factors and pro-inflammatory cytokines mediated by these pathways, including NF-κB, JNK/p38, NF/IL6, IRF, IFN-α/β, TNF-α, IL-2, IL-6, IL-8, and IL-10, was also be identified successfully. These suggest that TLR mechanisms are conserved from fish to mammals throughout vertebrate evolution. A putative draft of TLR signalling pathways in *L. japonicus *based on knowledge of TLR signalling in mammalian species was constructed (Figure 4). However, TLR signalling pathways in fish might be more intricate compared with those in mammalian species because of the novel TLRs (TLR14, TLR18, TLR21, and TLR23). An in-depth study of novel TLRs will improve understanding of fish-specific innate immunity in early vertebrates and even the complete evolutionary history of TLR-based innate immunity. DGE analysis revealed that TLR-1, -3, -13, -18, -21 and their signalling intermediates (Rac1, AKT, CASP8, IRAK1, TRAM, IRAK1, IKK alpha/beta, IRF7, and STAT1) were up- or down-regulated dramatically at different levels in the pathway upon bacterial challenge (P ≤ 0.01). This provides evidence that both conserved (TLR-1, -3, -13) and fish-specific (TLR-18, -21) TLR-based immunity participates in fish defence against bacterial challenge.

The innate immune system is generally believed to represent the evolutionarily ancient aspect of vertebrate immunity. As a representative of lower vertebrates, fish is suggested to possess stronger innate immune responses. However, fish adaptive immunity might be more primitive because of limited immunoglobulins and hallmark components necessary for adaptive immunity identified in this species [7]. In recent years, several hallmarks for T and B cells (TCR, BCR, CD3, CD4, and CD8), antigen presenting and processing molecules (MHCI, MHCII, and DC-SIGN/CD209), co-stimulatory factors (CD80/86, CD83, CD154, and CD40), and immunoglobulins (IgM, IgD, and IgZ/T) have been identified in teleost fish, thus providing preliminary evidence that the adaptive immune system might also be well-established in fish. However, the precise molecular and cellular bases and mechanisms underlying teleost adaptive immunity are still uncharacterised and require further immunogenetic studies. The present study successfully identified a large number of adaptive immune-relevant components homologous to those in higher vertebrates, providing abundant data sets for insights into the characterisation and origin of adaptive immunity in early vertebrates. Data sets imply that adaptive immunity in teleost fish seems to be much more complicated than previously believed. The basic components and signalling pathways necessary for adaptive immunity exist in fish, and a majority showed clear conservation between fish and mammals. For instance, T cell receptor (TCR) signalling pathways regulate T cell activation, one of the most important processes in adaptive immunity [51]. Majority of the four types of TCRs (TCR-α/β, -γδ) and numerous signalling transducers (Zap70, Lyn, LCK, SHP1, CD3, ITAM, LAT, Fyb, SLP-76, CBL, NCK, LAT, GRB2, CARMA1, NFAT, AP1, MALT1, and GRB2) discovered in humans and mammals can be identified in *L. japonicus*. DGE analysis showed that a number of TCR signalling pathway members, including TCR beta chain, Zap70, LCK, SHP1, CARMA1, Vav, NFAT, GRB2, MALT1, NCK, and Raf1, are induced significantly after bacterial challenge (P ≤ 0.01). These pathway members largely contribute to the proliferation and activation of T cells in mammals, thus suggesting that TCR signalling mechanisms underlying the T cell activation might be conserved between teleost fish and mammals. A putative draft of TCR signalling pathways based on knowledge of pathways known in mammals was constructed (Figure 5). Future studies on these pathways are expected to not only enrich current knowledge on fish immunology but also contribute to better understanding of the evolutionary history of adaptive immunity.

## Conclusions

This study investigated the transcriptome profile of bacteria-challenged *L. japonicus *using Solexa/Illumina RNA-seq and DGE deep sequencing technologies. The substantial amount of transcripts obtained provides a strong basis for future genomic research on marine fish and supports in-depth genome annotation in vertebrates. Globally identified immune-candidate genes, infection markers, and putative signalling pathways in *L. japonicus *revealed that the immune system of fish may be much more complex than previously believed. A considerable amount of immune-relevant genes and pathways in fish showed significant similarity to vertebrate models, suggesting that mechanisms underlying the innate and adaptive immunity in fish may be conserved in higher vertebrates. In addition, a large set of novel immune response genes that have never been linked previously to immune responses in other vertebrate systems indicate the existence of numerous fish-specific immune events during early evolution. This suggests that innate and adaptive immunity might be well established in teleost marine fish. Findings provide deep insight into the immunogenetics of fish species, which can be clinically applied in the therapy of fish diseases. They also contribute to a better understanding of the evolutionary history of innate and adaptive immunity from fish to mammals.

## Methods

### Experimental fish

One-year-old Japanese sea bass of both sexes, weighing 48.6 ± 2.5 g, were obtained from the fishery institute of Zhejiang, China. They were kept in running aerated seawater (salinity 30) at 25°C and fed with commercial pellet food at a daily ration of 0.7% body weight. All fish were maintained in the laboratory for at least two weeks prior to experimental use to allow for acclimatisation and evaluation of overall fish health. Only healthy fish, as determined by general appearance and level of activity, were used in the experiment.

### Bacterial strain

Wild-type marine fish-virulent *V harveyi *strain (96-915), a pathogen for bacterial septicaemia in *L. japonicas*, was maintained in the laboratory. It was cultured in Thiosulfate Citrate Bile Salts Sucrose at 27°C overnight. The desired number of cells was adjusted to 5 × 108 CFU/ml. Cells were inactivated with 5% formalin at 27°C overnight before thorough washing with sterile PBS (pH 7.0). They were re-suspended in PBS prior to use.

### Bacterial challenge and RNA preparation

Fish in the experimental groups were inoculated intraperitoneally with 0.2 ml of *V harveyi *at 1 × 10^8 ^CFU per fish. In parallel, fish in the control groups were administrated with 0.2 ml of mock PBS. Both groups were kept under conditions as described above. At seven days post-challenge, fish were sacrificed after anaesthesia, and tissues from the head kidney and spleen were collected. Tissue samples from 15 fishes were mixed for RNA preparation. Total RNA was isolated using a TRIzol reagent (Gibco BRL) following the manufacturer's instructions and treated with RNase free DNase I (Qiagen). RNA concentrations were measured using a spectrophotometer and integrity was ensured through analysis on a 1.5% (w/v) agarose gel.

### Sample Preparation for RNA-seq

After RNA extraction, poly-A-containing mRNAs were purified using oligo-dT-attached magnetic beads and fragmented into small pieces using divalent cations under elevated temperature. Cleaved RNA fragments were copied into first strand cDNA using reverse transcriptase and random primers. This was followed by second strand cDNA synthesis using DNA polymerase I and RNase H. These cDNA fragments underwent end repair process, addition of a single 'A' base, and ligation of adapters. Products were subsequently purified and amplified through PCR to create the final cDNA libraries.

### Transcriptome analysis

Transcriptome sequencing was conducted using Solexa/Illumina RNA-seq. Four fluorescently labelled nucleotides and a specialised polymerase were used to determine the clusters base by base in parallel. The 75-bp raw PE reads (submitted to GEO database, Association No. GSE21721) were generated by the Illumina Genome Analyzer II system. Raw reads were then assembled into non-redundant consensus sequences using Grape, tgicl, and CAP3 softwares [52,53]. All sequences were examined for possible sequencing errors. Adaptor sequences were trimmed using the Cross_Match software in the Phrap package http://www.phrap.org/[54]. Short sequences (< 100 bp in length) were removed using custom Perl program [55]. The resulting high-quality sequences were assembled into sequence contigs with the TGICL program, which creates an assembly using CAP3. Sequence homology searches were performed using local BLASTall programs against sequences in NCBI non-redundant (nr) protein database and the Swissprot database (E-value < 1e-10). Genes were tentatively identified according to the best hits against known sequences. Assembled consensus sequences were used to determine the GO term, COG term, and were analyzed further using KEGG.

### DGE-tag profiling

DGE analysis included sample preparation and sequencing. Sequence tag preparation was performed using the Digital Gene Expression Tag Profile Kit (Illumina) according to the manufacturer's instructions. Briefly, 6 μg total RNA was used for mRNA purification using oligo-dT magnetic bead adsorption and oligo-dT was used to guide reverse transcription for double-stranded cDNA synthesis. The generation of 5' ends of tags was done using endonuclease NlaIII, which recognizes and cuts off the CATG sites on cDNA. cDNA fragments with 3' ends were purified through magnetic bead precipitation, and Illumina adapter 1 was added to the 5' ends. The junction of Illumina adapter 1 and CATG site was the recognition site of MmeI, which cuts 17 bp downstream of the CATG site, producing tags with adapter 1. After removal of 3' fragments with magnetic bead precipitation, the 21-bp unique tags with adaptor 1 were purified and ligated to adaptor 2 to form a cDNA tag library. These adapter-ligated cDNA tags were enriched after 15 cycles of linear PCR amplification. The resulting 85-bp fragments were purified by 6% TBE polyacrylamide gel electrophoresis. Fragments were then digested and the single-chain molecules were fixed onto the Solexa Sequencing Chip (flowcell). Sequencing by synthesis was performed using the Illumina Genome Analyzer II system according to the manufacturer's protocols. Image analysis, base calling, generation of raw 17-bp tags, and tag counting were performed using the Illumina pipeline. Raw data (tag sequences) were deposited in the GEO database under submission number GSE21712.

### Aligning DGE tags to reference transcriptome data set

Clean tags and count number of DGE libraries from bacteria- and mock-challenged groups were collected and summarised using custom Bio-perl scripts. All tags were mapped to the reference transcriptome generated by RNA-seq. To monitor mapping events on both strands, both sense and complementary antisense sequences were included in the mapping process. Only perfect matches over the entire 21-bp length of the 17-bp tag plus the 4-bp NlaIII recognition site were allowed. This study was limited to tags that mapped to ORFs only and cannot show tags that mapped to mRNA with long 3'UTRs.

### Identification of differentially expressed genes

Rigorous algorithms were developed to identify differentially expressed genes between two samples. The correlation of the detected count numbers between parallel libraries was assessed statistically by calculating the Pearson correlation. In addition to the P value, FDR was manipulated to determine differentially expressed genes [56]. Assuming that R differentially expressed genes have been selected, S genes really show differential expression, whereas the other V genes are false positives. If the error ratio Q = V/R must remain below a cutoff (5%), FDR should not exceed 0.05. In this research, P ≤ 0.01, FDR ≤ 0.1, and the absolute value of log2Ratio≥1 were used as threshold to assess the significance of gene expression difference. More stringent criteria with smaller FDR and bigger fold-change values can be used to identify differentially expressed genes.

### Experimental validation

Representative consensus sequences with complete ORFs (hepcidin, Myf5, SNARE, syntaxin, and IL-8-like homologues) generated by RNA-seq were selected for experimental cloning and sequencing validation. The cDNAs of these genes were amplified by RT-PCR using the primers shown in Supplemental Table 6. All PCR products were purified using Gel Extraction Kit (Qiagen), cloned into pUCm-T vector (TaKaRa), and sequenced on MegaBACE 1000 Sequencer (GE) using the DYEnamic ET Dye Terminator Cycle Sequencing Kit (Pharmacia). Protein sequence alignments were generated using the Cluster W program (version 1.83). The phylogenies of protein sequences were estimated using MEGA 3.0 with the neighbour-joining method.

## Abbreviations

Most used abbreviations

ABCF: ATP-binding cassette subfamily F; AICDA: Activation-induced cytidine deaminase; AP-1: Activator protein 1; ASB: Ankyrin repeat and SOCS box protein; ASP: Apoptosis-stimulating of p53 protein; BTK: Bruton's tyrosine kinase; CAMP: Calmodulin-regulated spectrin-associated protein; CARD: Caspase recruitment domain; CBLB: E3 ubiquitin-protein ligase CBL-B; CDC: Cell division cycle; CDK: Cyclin-dependent kinase; CEBP: CCAAT/enhancer-binding protein; CFLAR: CASP8 and FADD-like apoptosis regulator; c-FOS: Cellular Proto-oncogene; CHUK: Conserved helix-loop-helix ubiquitous kinase; CNTFR: Ciliary neurotrophic factor receptor; CRADD: Death domain-containing protein CRADD; CRLF: Cytokine receptor-like factor; CSF: Macrophage/Granulocyte Colony-stimulating factor; CUB: First found in C1r/uEGF/bone morphogenetic protein; CYBB: Cytochrome b-245 heavy chain; CYFIP: Cytoplasmic FMR1 interacting protein; DEDD: Death effector domain-containing protein; DFD: Death-fold domain including CARD/DED/DEATH; DGCR: DiGeorge syndrome critical region; DMBT: Deleted in malignant brain tumors; DOCK: Dedicator of cytokinesis; DRAM: Damage-regulated autophagy modulator; Dscam: Down syndrome cell adhesion molecule; EBI: Epstein-Barr virus induced G-protein coupled receptor; EGF: Epidermal growth factor; EGR: Early growth response protein; EIF: Eukaryotic translation initiation factor; ELK: ETS domain-containing protein; EMAP: Echinoderm microtubule-associated protein; EMILIN: Elastin microfibril interfase located proteIN; ERCC: DNA-repair protein complementing XP-G cells homolog; FADD: Fas-associating death domain-containing protein; FAM: Family with sequence similarity; FGF: Fibroblast growth factor; FIMAC: Factor I membrane attack complex; FLT: Fms-related tyrosine kinase; FND: Fibronectin type III domain-containing protein; GALNAC4S-6ST: N-acetylgalactosamine 4-sulfate 6-O-sulfotransferase GALNAC4S6ST; GATA: Trans-acting T-cell specific transcription factor; GDF: Growth/differentiation factor; GFI: Growth factor independent; GPR: G-protein coupled receptor; HELLS: Lymphoid-specific helicase encoded; HLA: Human leukocyte antigen; HMG: High mobility group protein; HSP: Heat shock protein; HTR: HEAT repeat-containing protein; ICAM: Intercellular adhesion molecule; ICE: Interleukin Catalytic Enzyme; IFRD: Interferon-related developmental regulator; IGBP: Immunoglobulin-binding protein; IKKA: Inhibitor of nuclear factor κ-B kinase subunit α; IMPDH/IMDH: Inosine-5'-monophosphate dehydrogenase; IRAK: Interleukin-1 receptor-associated kinase; IRG: Immune-responsive gene; ITCH: Itchy homolog E3; JAK: Janus kinase; JAG: Protein jagged-1; JNK: c-Jun N-terminal kinase; KI13B: Kinesin-like protein KIF13B; KLF: Krueppel-like factor; LAG: Lymphocyte activation gene; LIFR: Leukemia inhibitory factor receptor; LRP: Prolow-density lipoprotein receptor-related protein; LRR: Leucine-rich repeat; MACPE: Membrane attack complex/perforin; MAF: Macrophage activating factor; MALT: Mucosa-associated lymphoma translocation protein; MAPK: Mitogen-activated protein kinase; MASP: Mannose binding lectin associated serine protease; MBL: Mannose binding lectin; MCA: Multisynthetase complex auxiliary component; MHC: Major histocompatibility complex; MIF: Macrophage migration inhibitory factor; Myd88: Myeloid differentiation primary response gene 88; NALP: NACHT; LRR and PYD domains-containing protein; NCK: Cytoplasmic protein; NF κ: Nuclear factor κ; NFAT: Nuclear factors of activated T cells; NOD: Nucleotide-binding oligomerization domain; NOS: Nitric oxide synthase; NR2C: Nuclear receptor subfamily 2 group C; OSGI: Oxidative stress-induced growth inhibitor; PAMP: Pathogen-associated molecular pattern; PARC: p53-associated parkin-like cytoplasmic protein; PAWR: PRKC apoptosis WT1 regulator protein; PCGF: Polycomb group RING finger protein; PEA15: Phosphoprotein enriched in astrocytes 15 gene; PGRP: Peptidoglycan recognition protein; PHLA: Pleckstrin homology-like domain family A; PIGR: Polymeric immunoglobulin receptor; PPARA: Peroxisome proliferator-activated receptor α; PRR: PAMP recognition receptor; PSMA: Proteasome subunit α type; PSMB: Proteasome subunit β type; PSMC: Protease 26 S subunit; ATPase; PSMD: Proteasome 26 S subunit; non-ATPase; PSME: Proteasome activator complex subunit; PTAFR: Platelet-activating factor receptor; PYCARD: PYD and CARD domain containing protein; PYD: Domain in pyrin; RAC: Ras-related C3 botulinum toxin substrate; REL: C-Rel proto-oncogene protein; RFX5: Regulatory factor × 5; RGC: Response gene to complement protein; RGS: Regulator of G-protein signaling; RHOA: Ras homolog gene family A; RIPK: Receptor-interacting serine/threonine-protein kinase; SARM: SAM and TIR containing; SCYE: Small inducible cytokine subfamily E; SFTPD/SP-D: Surfactant; pulmonary-associated protein D; SIGIRR: Single Ig IL-1-related receptor; SLC20A-1/2: Sodium-dependent phosphate transporter 1/2; SMAD: Mothers against decapentaplegic; SNED: Sushi nidogen and EGF-like domain-containing protein; SOCS: Suppressor of cytokine signaling; TANK: TRAF associated NF-κ-B activator; TAPBP: TAP binding protein; TBX: T-box transcription factor; TGFA: Transforming growth factor -associated protein; TICAM: Toll-like receptor adapter molecule; TIRAP: Toll/interleukin-1 receptor domain-containing adapter protein; TMED: Transmembrane emp24 domain-containing protein; TNF(TNR): Tumor necrosis factor (receptor); TOLLIP: Toll-interacting protein; TPR: Tetratrico peptide repeat; TRADD: TNFRSF1A-associated death domain protein; TRAF: TNF-receptor-associated factor; TRIM: Tripartite motif-containing protein; TSP1: Thrombospondin type 1 repeats; TYK: Tyrosine-protein kinase; UBE: Ubiquitin-conjugating enzyme; UCRP/CRP: Ubiquitin cross-reactive protein; VEGF: Vascular endothelial growth factor; WAS: Wiskott-Aldrich syndrome; WSC: Cell wall integrity and stress response component; WWP: WW domain-containing proteins; XPA: DNA-repair protein complementing XP-A cells.

## Authors' contributions

LXX and DH conceived and designed the study, participated in the bioinformatics analysis, and drafted the manuscript. WRD and YWZ performed the experiments and designed the tables. JZS conceptualized the project, reviewed the manuscript, and provided guidance. All authors read and approved the final manuscript.

## Acknowledgements

We acknowledge the Beijing Genomics Institute at Shenzhen for its assistance in original data processing and related bioinformatics analysis. We are thankful to professor Guo-liang Wang of Ningbo University for providing us with *Vibrio harveyi *culture. We would also like to thank Guang-ping Liu, Hui-hui Liu, and Jian-qiu Zou for their help in data and figure processing. This work was supported by grants from Hi-Tech Research and Development Program of China (863) (2008AA09Z409), the National Basic Research Program of China (973) (2006CB101805), the National Natural Science Foundation of China (30871936, 30571423), and the Science and Technology Foundation of Zhejiang Province (2006C12038, 2006C23045, 2006C12005, 2007C12011).

## Supplementary Material

Additional file 1 **Table S1: Details on immune-relevant genes/homologues in *L. japonicas***. Click here for file

Additional file 2 **Table S2: Summary of tag mapping in DGE analysis for experimental and control groups**. Click here for file

Additional file 3 **Figure S1: Tag abundance for mock- (A) and bacteria- (B) challenged group**. Normalised tag copy number was calculated by dividing tag counts for each gene with the total number of tags generated for each library and are presented per one million transcripts. PM and 1 MM stand for perfect match and 1 miss match, respectively. Click here for file

Additional file 4 **Table S3: Summary of differentially expressed CD-containing consensus sequences**. Click here for file

Additional file 5 **Table S4: Summary of putative novel immune/stress response consensus**. Each consensus was analyzed using ProDom, SignalP, and TMHMM programs. Click here for file

Additional file 6 **Table S5: Primers used for validation analysis**. Click here for file

Additional file 7 **Figure S2: Clustal W analysis for all IL-8-like CXC chemokines across all vertebrates**. Click here for file

Additional file 8 **Figure S3: Phylogenetic analysis for all IL-8-like CXC chemokines across all vertebrates**. A phylogenetic tree was constructed using the maximum likelihood method to show the relationship between L. japonicus IL-8 and other known vertebrate IL-8-like CXC-chemokines. Local bootstrap percentages were obtained after 10000 replications. Click here for file

## References

[B1] Kocher TD Adaptive evolution and explosive speciation: the cichlid fish model Nat Rev Genet 2004 5 4 288 298 10.1038/nrg1316 15131652

[B2] Venkatesh B Evolution and diversity of fish genomes Curr Opin Genet Dev 2003 13 6 588 592 10.1016/j.gde.2003.09.001 14638319

[B3] Kasahara M Naruse K Sasaki S Nakatani Y Qu W Ahsan B Yamada T Nagayasu Y Doi K Kasai Y The medaka draft genome and insights into vertebrate genome evolution Nature 2007 447 7145 714 719 10.1038/nature05846 17554307

[B4] Peatman E Liu Z Evolution of CC chemokines in teleost fish: a case study in gene duplication and implications for immune diversity Immunogenetics 2007 59 8 613 623 10.1007/s00251-007-0228-4 17541578

[B5] Zou J Tafalla C Truckle J Secombes CJ Identification of a second group of type I IFNs in fish sheds light on IFN evolution in vertebrates J Immunol 2007 179 6 3859 3871 17785823 10.4049/jimmunol.179.6.3859

[B6] Jin HJ Shao JZ Xiang LX Wang H Sun LL Global identification and comparative analysis of SOCS genes in fish: insights into the molecular evolution of SOCS family Mol Immunol 2008 45 5 1258 1268 10.1016/j.molimm.2007.09.015 18029016

[B7] Flajnik MF Kasahara M Origin and evolution of the adaptive immune system: genetic events and selective pressures Nat Rev Genet 2010 11 1 47 59 10.1038/nrg2703 19997068 PMC3805090

[B8] Leulier F Lemaitre B Toll-like receptors--taking an evolutionary approach Nat Rev Genet 2008 9 3 165 178 10.1038/nrg2303 18227810

[B9] Hegedus Z Zakrzewska A Agoston VC Ordas A Racz P Mink M Spaink HP Meijer AH Deep sequencing of the zebrafish transcriptome response to mycobacterium infection Mol Immunol 2009 46 15 2918 2930 10.1016/j.molimm.2009.07.002 19631987

[B10] Stockhammer OW Zakrzewska A Hegedus Z Spaink HP Meijer AH Transcriptome profiling and functional analyses of the zebrafish embryonic innate immune response to Salmonella infection J Immunol 2009 182 9 5641 5653 10.4049/jimmunol.0900082 19380811

[B11] Wang Z Gerstein M Snyder M RNA-Seq: a revolutionary tool for transcriptomics Nat Rev Genet 2009 10 1 57 63 10.1038/nrg2484 19015660 PMC2949280

[B12] Anisimov SV Serial Analysis of Gene Expression (SAGE): 13 years of application in research Curr Pharm Biotechnol 2008 9 5 338 350 10.2174/138920108785915148 18855686

[B13] Mortazavi A Williams BA McCue K Schaeffer L Wold B Mapping and quantifying mammalian transcriptomes by RNA-Seq Nat Methods 2008 5 7 621 628 10.1038/nmeth.1226 18516045 PMC13303166

[B14] Sultan M Schulz MH Richard H Magen A Klingenhoff A Scherf M Seifert M Borodina T Soldatov A Parkhomchuk D A global view of gene activity and alternative splicing by deep sequencing of the human transcriptome Science 2008 321 5891 956 960 10.1126/science.1160342 18599741

[B15] Nagalakshmi U Wang Z Waern K Shou C Raha D Gerstein M Snyder M The transcriptional landscape of the yeast genome defined by RNA sequencing Science 2008 320 5881 1344 1349 10.1126/science.1158441 18451266 PMC2951732

[B16] Morozova O Marra MA Applications of next-generation sequencing technologies in functional genomics Genomics 2008 92 5 255 264 10.1016/j.ygeno.2008.07.001 18703132

[B17] t Hoen PA Ariyurek Y Thygesen HH Vreugdenhil E Vossen RH de Menezes RX Boer JM van Ommen GJ den Dunnen JT Deep sequencing-based expression analysis shows major advances in robustness, resolution and inter-lab portability over five microarray platforms Nucleic Acids Res 2008 36 21 e141 10.1093/nar/gkn705 18927111 PMC2588528

[B18] Wang B Guo G Wang C Lin Y Wang X Zhao M Guo Y He M Zhang Y Pan L Survey of the transcriptome of Aspergillus oryzae via massively parallel mRNA sequencing Nucleic Acids Res 2010 in press 20392818 10.1093/nar/gkq256 PMC2926611

[B19] Levin JZ Berger MF Adiconis X Rogov P Melnikov A Fennell T Nusbaum C Garraway LA Gnirke A Targeted next-generation sequencing of a cancer transcriptome enhances detection of sequence variants and novel fusion transcripts Genome Biol 2009 10 10 R115 10.1186/gb-2009-10-10-r115 19835606 PMC2784330

[B20] Tang F Barbacioru C Nordman E Li B Xu N Bashkirov VI Lao K Surani MA RNA-Seq analysis to capture the transcriptome landscape of a single cell Nat Protoc 2010 5 3 516 535 10.1038/nprot.2009.236 20203668 PMC3847604

[B21] Asmann YW Klee EW Thompson EA Perez EA Middha S Oberg AL Therneau TM Smith DI Poland GA Wieben ED 3' tag digital gene expression profiling of human brain and universal reference RNA using Illumina Genome Analyzer BMC Genomics 2009 10 531 10.1186/1471-2164-10-531 19917133 PMC2781828

[B22] Zhang G Guo G Hu X Zhang Y Li Q Li R Zhuang R Lu Z He Z Fang X Deep RNA sequencing at single base-pair resolution reveals high complexity of the rice transcriptome Genome Res 2010 20 646 654 10.1101/gr.100677.109 20305017 PMC2860166

[B23] David JP Coissac E Melodelima C Poupardin R Riaz MA Chandor-Proust A Reynaud S Transcriptome response to pollutants and insecticides in the dengue vector Aedes aegypti using next-generation sequencing technology BMC Genomics 2010 11 1 216 10.1186/1471-2164-11-216 20356352 PMC2867825

[B24] Liu S Li D Li Q Zhao P Xiang Z Xia Q MicroRNAs of Bombyx mori identified by Solexa sequencing BMC Genomics 2010 11 148 10.1186/1471-2164-11-148 20199675 PMC2838851

[B25] Yassour M Kaplan T Fraser HB Levin JZ Pfiffner J Adiconis X Schroth G Luo S Khrebtukova I Gnirke A Ab initio construction of a eukaryotic transcriptome by massively parallel mRNA sequencing Proc Natl Acad Sci USA 2009 106 9 3264 3269 10.1073/pnas.0812841106 19208812 PMC2638735

[B26] Veitch NJ Johnson PC Trivedi U Terry S Wildridge D MacLeod A Digital gene expression analysis of two life cycle stages of the human-infective parasite, Trypanosoma brucei gambiense reveals differentially expressed clusters of co-regulated genes BMC Genomics 2010 11 124 10.1186/1471-2164-11-124 20175885 PMC2837033

[B27] Morrissy AS Morin RD Delaney A Zeng T McDonald H Jones S Zhao Y Hirst M Marra MA Next-generation tag sequencing for cancer gene expression profiling Genome Res 2009 19 10 1825 1835 10.1101/gr.094482.109 19541910 PMC2765282

[B28] Wang Y Brahmakshatriya V Zhu H Lupiani B Reddy SM Yoon BJ Gunaratne PH Kim JH Chen R Wang J Identification of differentially expressed miRNAs in chicken lung and trachea with avian influenza virus infection by a deep sequencing approach BMC Genomics 2009 10 512 10.1186/1471-2164-10-512 19891781 PMC2777939

[B29] Chen X Li Q Wang J Guo X Jiang X Ren Z Weng C Sun G Wang X Liu Y Identification and characterization of novel amphioxus microRNAs by Solexa sequencing Genome Biol 2009 10 7 R78 10.1186/gb-2009-10-7-r78 19615057 PMC2728532

[B30] Xie ZY Hu CQ Zhang LP Chen C Ren CH Shen Q Identification and pathogenicity of Vibrio ponticus affecting cultured Japanese sea bass, Lateolabrax japonicus (Cuvier in Cuvier and Valenciennes) Letters in Applied Microbiology 2007 45 1 62 67 10.1111/j.1472-765X.2007.02141.x 17594462

[B31] Austin B Zhang XH Vibrio harveyi: a significant pathogen of marine vertebrates and invertebrates Lett Appl Microbiol 2006 43 2 119 124 10.1111/j.1472-765X.2006.01989.x 16869892

[B32] Iseli C Jongeneel CV Bucher P ESTScan: a program for detecting, evaluating, and reconstructing potential coding regions in EST sequences Proc Int Conf Intell Syst Mol Biol 1999 138 148 10786296

[B33] Ye J McGinnis S Madden TL BLAST: improvements for better sequence analysis Nucleic Acids Res 2006 34 Web Server W6 9 10.1093/nar/gkl164 16845079 PMC1538791

[B34] Ye J Fang L Zheng H Zhang Y Chen J Zhang Z Wang J Li S Li R Bolund L WEGO: a web tool for plotting GO annotations Nucleic Acids Res 2006 34 Web Server W293 297 10.1093/nar/gkl031 16845012 PMC1538768

[B35] Tatusov RL Galperin MY Natale DA Koonin EV The COG database: a tool for genome-scale analysis of protein functions and evolution Nucleic Acids Res 2000 28 1 33 36 10.1093/nar/28.1.33 10592175 PMC102395

[B36] Kanehisa M Araki M Goto S Hattori M Hirakawa M Itoh M Katayama T Kawashima S Okuda S Tokimatsu T KEGG for linking genomes to life and the environment Nucleic Acids Res 2008 36 Database D480 484 18077471 10.1093/nar/gkm882 PMC2238879

[B37] Rassaf T Kleinbongard P Kelm M The L-arginine nitric oxide pathway: avenue for a multiple-level approach to assess vascular function Biol Chem 2006 387 10-11 1347 1349 10.1515/BC.2006.168 17081105

[B38] Ewing B Hillier L Wendl MC Green P Base-calling of automated sequencer traces using phred. I. Accuracy assessment Genome Res 1998 8 3 175 185 9521921 10.1101/gr.8.3.175

[B39] Ewing B Green P Base-calling of automated sequencer traces using phred. II. Error probabilities Genome Res 1998 8 3 186 194 9521922

[B40] Audic S Claverie JM The significance of digital gene expression profiles Genome Res 1997 7 10 986 995 9331369 10.1101/gr.7.10.986

[B41] Li R Yu C Li Y Lam TW Yiu SM Kristiansen K Wang J SOAP2: an improved ultrafast tool for short read alignment Bioinformatics 2009 25 15 1966 1967 10.1093/bioinformatics/btp336 19497933

[B42] Bru C Courcelle E Carrere S Beausse Y Dalmar S Kahn D The ProDom database of protein domain families: more emphasis on 3D Nucleic Acids Res 2005 33 Database D212 215 15608179 10.1093/nar/gki034 PMC539988

[B43] Bendtsen JD Nielsen H von Heijne G Brunak S Improved prediction of signal peptides: SignalP 3.0 J Mol Biol 2004 340 4 783 795 10.1016/j.jmb.2004.05.028 15223320

[B44] Chen Y Yu P Luo J Jiang Y Secreted protein prediction system combining CJ-SPHMM, TMHMM, and PSORT Mamm Genome 2003 14 12 859 865 10.1007/s00335-003-2296-6 14724739

[B45] Borchardt T Looso M Bruckskotten M Weis P Kruse J Braun T Analysis of newly established EST databases reveals similarities between heart regeneration in newt and fish BMC Genomics 2010 11 4 10.1186/1471-2164-11-4 20047682 PMC2823690

[B46] Lo L Zhang Z Hong N Peng J Hong Y 3640 unique EST clusters from the medaka testis and their potential use for identifying conserved testicular gene expression in fish and mammals PLoS One 2008 3 12 e3915 10.1371/journal.pone.0003915 19104663 PMC2603314

[B47] Lin AF Xiang LX Wang QL Dong WR Gong YF Shao JZ The DC-SIGN of zebrafish: insights into the existence of a CD209 homologue in a lower vertebrate and its involvement in adaptive immunity J Immunol 2009 183 11 7398 7410 10.4049/jimmunol.0803955 19890038

[B48] Gong YF Xiang LX Shao JZ CD154-CD40 interactions are essential for thymus-dependent antibody production in zebrafish: insights into the origin of costimulatory pathway in helper T cell-regulated adaptive immunity in early vertebrates J Immunol 2009 182 12 7749 7762 10.4049/jimmunol.0804370 19494299

[B49] Meijer AH Gabby Krens SF Medina Rodriguez IA He S Bitter W Ewa Snaar-Jagalska B Spaink HP Expression analysis of the Toll-like receptor and TIR domain adaptor families of zebrafish Mol Immunol 2004 40 11 773 783 10.1016/j.molimm.2003.10.003 14687934

[B50] Oshiumi H Tsujita T Shida K Matsumoto M Ikeo K Seya T Prediction of the prototype of the human Toll-like receptor gene family from the pufferfish, Fugu rubripes, genome Immunogenetics 2003 54 11 791 800 12618912 10.1007/s00251-002-0519-8

[B51] Meeker ND Smith AC Frazer JK Bradley DF Rudner LA Love C Trede NS Characterization of the zebrafish T cell receptor beta locus Immunogenetics 62 1 23 29 10.1007/s00251-009-0407-6 20054533

[B52] Pertea G Huang X Liang F Antonescu V Sultana R Karamycheva S Lee Y White J Cheung F Parvizi B TIGR Gene Indices clustering tools (TGICL): a software system for fast clustering of large EST datasets Bioinformatics 2003 19 5 651 652 10.1093/bioinformatics/btg034 12651724

[B53] Huang X Madan A CAP3: A DNA sequence assembly program Genome Res 1999 9 9 868 877 10.1101/gr.9.9.868 10508846 PMC310812

[B54] de la Bastide M McCombie WR Assembling genomic DNA sequences with PHRAP Curr Protoc Bioinformatics 2007 Chapter 11 Unit11 14 18428783 10.1002/0471250953.bi1104s17

[B55] Stajich JE Block D Boulez K Brenner SE Chervitz SA Dagdigian C Fuellen G Gilbert JG Korf I Lapp H The Bioperl toolkit: Perl modules for the life sciences Genome Res 2002 12 10 1611 1618 10.1101/gr.361602 12368254 PMC187536

[B56] Benjamini Y Drai D Elmer G Kafkafi N Golani I Controlling the false discovery rate in behavior genetics research Behav Brain Res 2001 125 1-2 279 284 10.1016/S0166-4328(01)00297-2 11682119

