# Supplementary figures and images for: Deep sequencing-based transcriptome profiling analysis of bacteria-challenged Lateolabrax japonicus reveals insight into the immune-relevant genes in marine fish
Source: BMC Genomics. 2010 Aug 13;11:472. doi: 10.1186/1471-2164-11-472 (PMC3091668; doi:10.1186/1471-2164-11-472)

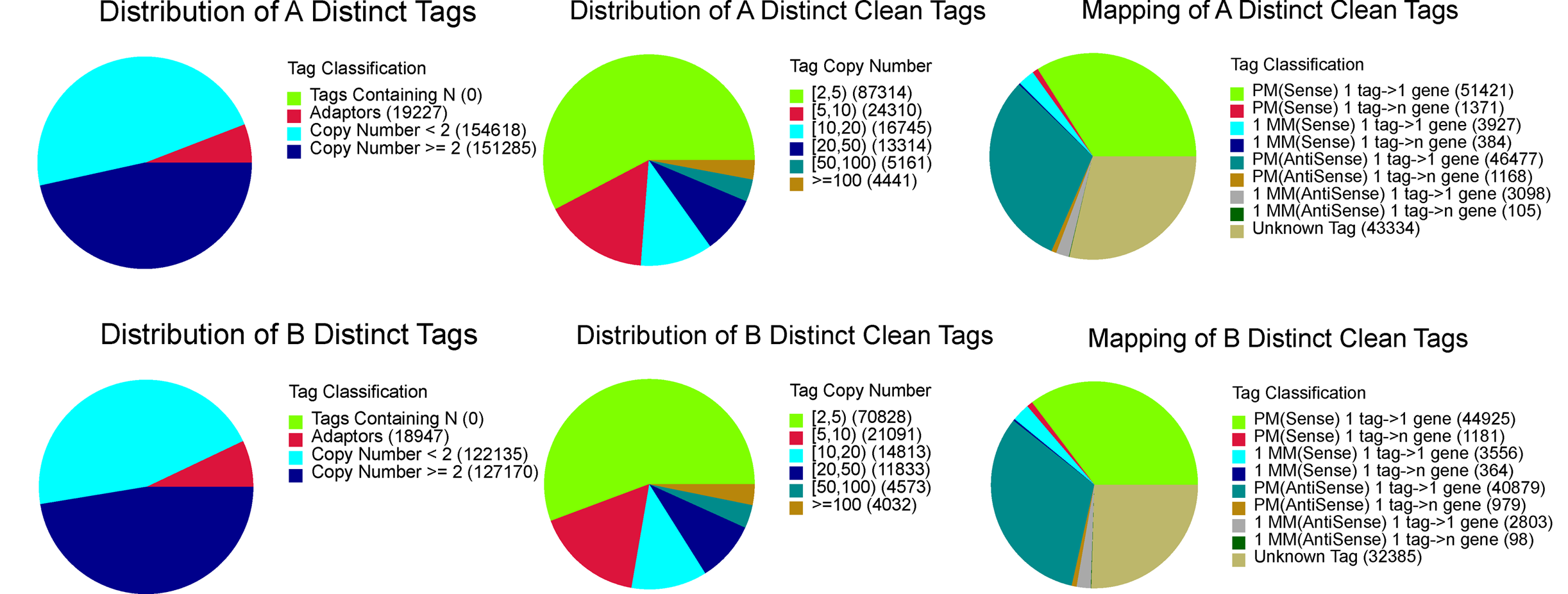

Supplement: Additional file 3 — Figure S1: Tag abundance for mock- (A) and bacteria- (B) challenged group. Normalised tag copy number was calculated by dividing tag counts for each gene with the total number of tags generated for each library and are presented per one million transcripts. PM and 1 MM stand for perfect match and 1 miss match, respectively. [file 1471-2164-11-472-S3.TIFF]

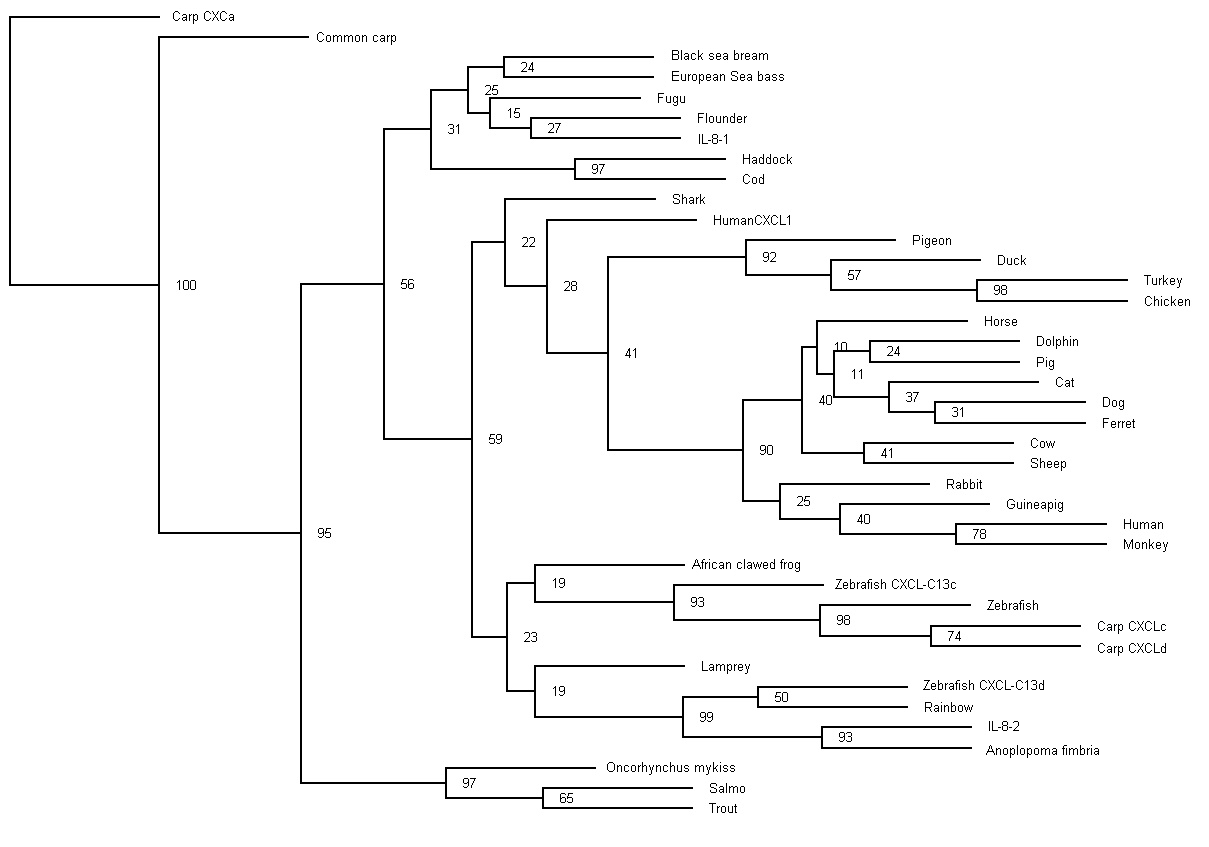

Supplement: Additional file 8 — Figure S3: Phylogenetic analysis for all IL-8-like CXC chemokines across all vertebrates. A phylogenetic tree was constructed using the maximum likelihood method to show the relationship between L. japonicus IL-8 and other known vertebrate IL-8-like CXC-chemokines. Local bootstrap percentages were obtained after 10000 replications. [file 1471-2164-11-472-S8.TIFF]
