# Supplementary material for: Deep sequencing-based transcriptome profiling analysis of bacteria-challenged Lateolabrax japonicus reveals insight into the immune-relevant genes in marine fish
Source: BMC Genomics. 2010 Aug 13;11:472. doi: 10.1186/1471-2164-11-472 (PMC3091668; doi:10.1186/1471-2164-11-472)
Supplement: Additional file 7 — Figure S2: Clustal W analysis for all IL-8-like CXC chemokines across all vertebrates. [file 1471-2164-11-472-S7.PDF]

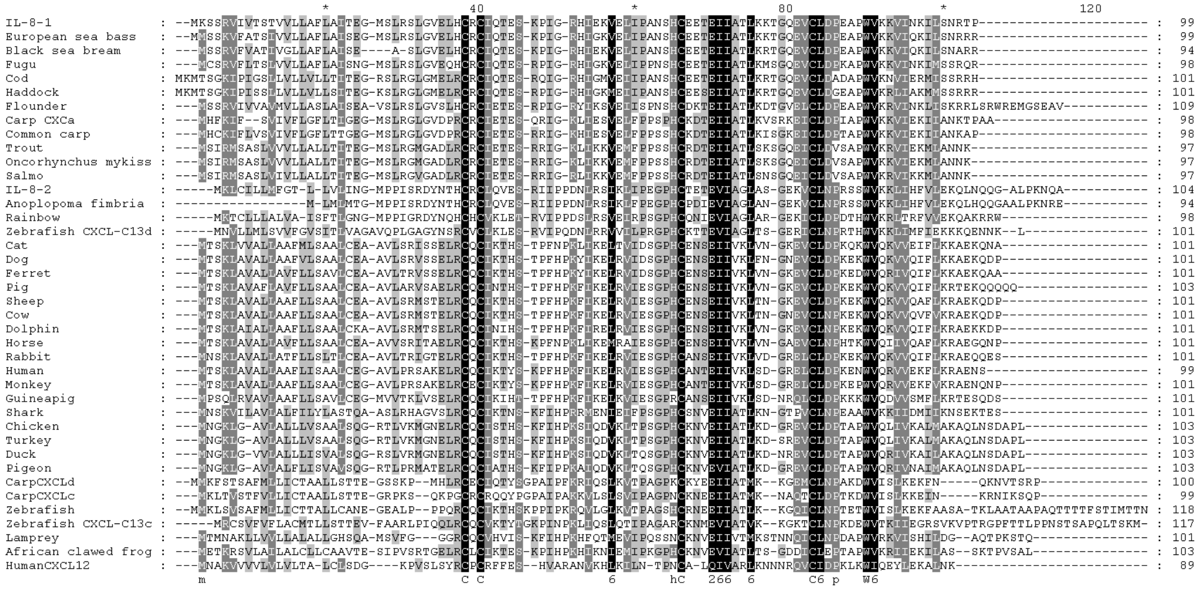

Figure 2. Amino acid alignment of *Lateolabrax japonicus* IL-8 with known vertebrate IL-8 homologues. The conserved CXC motif is shaded and the ELR motif marked in bold. Hyphens indicate gaps. Identical residues are marked by asterisks and highly and moderately conserved residues are shown below the alignment by colons and dots respectively. The database accession numbers of the IL-8 reported aligned sequences and phylogenetic tree are: Human IL-8, P10145; Human CXCL12, CAG29279; Monkey IL-8, P51495; Cat IL-8, Q9XSX5; Dog IL-8, P41324; Sheep IL-8, P36925; Cow IL-8, P79255; Pig IL-8, P26894; Guinea pig IL-8, P49113; Horse IL-8, O62812; Rabbit IL-8, P19874; Ferret IL-8, BAF56573; Dolphin IL-8, Q7YRB5; Chicken EMF-1, P08317; Pigeon IL-8, ABD49206; Turkey IL-8, ABD49207; Duck IL-8, BAF02383; Haddock IL-8, AJ566335; Cod IL-8, CAD59734; Shark IL-8, AB063299; Black sea bream IL-8, DQ000611; Fugu IL-8, AB125645; Flounder IL-8, AF216646; Trout IL-8, AJ279069; Zebrafish CXCL-C13c (zCXCL-chr13c), BAF98258; Zebrafish CXCL-C13d (zCXCL-13d), BAF98259; Zebrafish (Zf) EH441857, EH441857; Carp CXCa, AJ421443; Carp CXCLc, EC394283; Carp CXCLd, EX881663 and Lamprey IL-8, AJ231072; Oncorhynchus miss,AAO25640.1; Common carp, Q1PBV7; Salmo, B5X6N7; Rainbow, C1BLE9; African clawed frog, A1A632; Atlantic salmon, B5X6N7; Anoplopoma fimbria, ACQ58275.1.
